# Novel Germline Mutations in a Cohort of Men with Familial Prostate Cancer

**DOI:** 10.3390/cancers14153623

**Published:** 2022-07-26

**Authors:** Romy Mondschein, Damien Bolton, David Clouston, James Dowty, Liam Kavanagh, Declan Murphy, Prudence Scott, Renea A. Taylor, Heather Thorne

**Affiliations:** 1 kConFab, Research Division, Peter MacCallum Cancer Centre, East Melbourne 3002, Australia; renea.taylor@monash.edu; 2 Austin Health, Urology, Heidelberg, Melbourne 3084, Australia; damienmbolton@gmail.com; 3 TissuPath, Glen Waverley, Melbourne 3150, Australia; david.clouston@tissupath.com.au; 4 Melbourne School of Population and Global Health, The University of Melbourne, Parkville, Melbourne 3010, Australia; jdowty@unimelb.edu.au; 5 The Northern Hospital, Epping 3076, Australia; practice@brunurology.com.au; 6 Sir Peter MacCallum Department of Oncology, The University of Melbourne, Parkville, Melbourne 3010, Australia; declan.murphy@petermac.org; 7 Monash Health Familial Cancer Centre, Clayton 3168, Australia; prudence.scott@monashhealth.org; 8 Department of Physiology and Biomedicine Discovery Institute, Monash University, Melbourne 3800, Australia

**Keywords:** prostate cancer, germline, hereditary, mutations, pathogenic variants

## Abstract

**Simple Summary:**

In this study, we identified germline mutations that contribute to prostate cancer development in men who have multiple relatives with prostate cancer (and other cancers). We correlated the genetic mutations found in each patient with the resulting prostate cancer characteristics. Mutations found in *ATM* and *CHEK2* genes were associated with aggressive prostate cancer. More data is needed to confirm the prevalence and impact of these germline mutations in prostate cancer.

**Abstract:**

***Background:*** Germline mutations in *BRCA2* are associated with aggressive prostate cancer. Additional information regarding the clinical phenotype of germline pathogenic variants in other prostate cancer predisposition genes is required. Clinical testing has been limited by evidence, further restricting knowledge of variants that contribute to prostate cancer development. ***Objective:*** Prostate cancer patients who were first- and second-degree relatives from multi-case prostate cancer families underwent a gene panel screen to identify novel (non-*BRCA*) germline pathogenic variants in cancer predisposition genes and define clinical phenotypes associated with each gene. ***Methods:*** The germline genomic DNA (gDNA) of 94 index cases with verified prostate cancer from families with a minimum of two verified prostate cancer cases was screened with an 84-cancer-gene panel. Families were recruited for multi-case breast/ovarian cancer (*n* = 66), or multi-case prostate cancer (*n* = 28). Prostate cancer characteristics associated with each gene were compared with prostate cancer cases of confirmed non-mutation carriers (*BRCAX*), also from multi-case prostate cancer families (*n* = 111), and with data from the Prostate Cancer Outcomes Registry (PCOR). ***Results:*** Ninety-four prostate cancer index cases underwent gene panel testing; twenty-two index cases (22/94; 23%) were found to carry a class 4–5 (C4/5) variant. Six of twenty-two (27%) variants were not clinically notifiable, and seven of twenty-two (31.8%) variants were in *BRCA1/2* genes. Nine of twenty-two (40.9%) index cases had variants identified in *ATM* (*n* = 4), *CHEK2* (*n* = 2) and *HOXB13G84* (*n* = 3); gDNA for all relatives of these nine cases was screened for the corresponding familial variant. The final cohort comprised 15 confirmed germline mutation carriers with prostate cancer (*ATM n* = 9, *CHEK2 n* = 2, *HOXB13G84 n* = 4). *ATM* and *CHEK2*-associated cancers were D’Amico intermediate or high risk, comparable to our previously published *BRCA2* and *BRCAX* prostate cancer cohort. *HOXB13G84* carriers demonstrated low- to intermediate-risk prostate cancer. In the *BRCAX* cohort, 53.2% of subjects demonstrated high-risk disease compared with 25% of the PCOR cohort. ***Conclusions:*** *ATM* and *CHEK2* germline mutation carriers and the *BRCAX* (confirmed non-mutation carriers) cohort demonstrated high risk disease compared with the general population. Targeted genetic testing will help identify men at greater risk of prostate-cancer-specific mortality. Data correlating rare variants with clinical phenotype and familial predisposition will strengthen the clinical validity and utility of these results and establish these variants as significant in prostate cancer detection and management.

## 1. Introduction

Efforts to identify groups of men at risk of developing clinically significant prostate cancer indicate that those with a family history are at significantly higher risk compared with the general population [1,2,3]. For these individuals and their families, discovering the genetic aetiology of hereditary prostate cancer and clinical phenotype associated with each variant has broad clinical utility. Gene-specific risk stratification, targeted screening, early intervention and novel therapeutic targets are realisable outcomes. These advances are likely to reduce the burden on health systems and individuals compared with managing disease at advanced stages [4,5]. Recent studies have highlighted the benefits and feasibility of dedicated clinics to serve high-risk prostate cancer patients with pathogenic mutations in PC susceptibility genes, and clinics dedicated to identifying these high-risk individuals in a streamlined fashion [6,7]. Modified protocols for *BRCA2* mutation carriers have resulted in improved clinical outcomes [8,9]. The potential for treatment response to poly (ADP-ribose) polymerase (PARP) inhibitors in mutation carriers with prostate cancer has been strengthening support for screening of relevant familial cohorts [10,11]. 

Gene panel sequencing is increasingly utilised to predict cancer risk in patients from multi-case cancer families. However, in the familial prostate cancer setting, the clinical utility of panel testing is limited by our current understanding of which germline pathogenic variants are associated with aggressive disease. Although research conducted in patients with prostate cancer unselected for family history, stage of disease or age at diagnosis has contributed to prevalence estimates of mutations [12], a lack of comprehensive clinical records impedes the clinical associations required to inform clinical management. In this study, we aim to identify novel germline variants in prostate cancer predisposition genes and describe their associated clinical phenotype in the setting of multi-case prostate cancer families and multi-case breast/ovarian cancer families. We provide evidence that novel germline mutations in *ATM* and *CHEK2* are associated with aggressive prostate cancer. This finding will contribute evidence to support broader genetic screening and development of gene-targeted protocols, with the aim of improving outcomes in these high-risk prostate cancer patients. 

## 2. Patients and Methods

### 2.1. Ethics Statement

Signed consent was obtained from all participants on recruitment to the Kathleen Cunningham Familial Cancer Consortium (kConFab) (www.kconfab.org (accessed on 31 March 2021)). This project was approved by the Peter MacCallum Cancer Centre, Melbourne, Australia, Human Research Ethics Committee, protocol #97/27. 

### 2.2. Study Population

Families containing at least two male first- or second-degree relatives with verified prostate cancer were identified from kConFab [13]. kConFab recruitment of these families was from surgical and genetics services on the basis of multi-case prostate cancer (*n* = 28) or multi-case breast/ovarian cancer (*n* = 66). Males were eligible for inclusion if (a) their prostate cancer could be verified with a pathology report and/or doctor’s notes, (b) treatment reports were available and (c) a gDNA sample was available for mutation testing. An index case (*n* = 94) was selected for mutation testing from each family based on high-grade pathological features and/or youngest age at diagnosis. All cancers were prostatic adenocarcinoma with index cases diagnosed between 1994 and 2019. Affected females in the breast/ovarian cancer families had previously undergone a seven-cancer-gene panel screening (*BRCA1*, *BRCA2*, *ATM 7271*, *PALB2*, *TP53*, *PTEN*, *CHEK2* *1100delC*) prior to this study, with no C4/5 variant identified. 

### 2.3. Clinical and Pathological Data

Age, stage at diagnosis and prostate-specific antigen (PSA) immediately prior to biopsy was recorded. Gleason score (GS) at diagnosis (first biopsy) and radical prostatectomy were converted to ISUP Grade Group (GG) [14] for analysis. Treatment modality, cause and date of death and presence of other primary verified malignancies were recorded. D’Amico risk stratification [15] was derived from PSA, GG and stage at diagnosis. 

Formalin-fixed paraffin-embedded archival 5μm prostate cancer (biopsy or prostatectomy) tissue specimens were haematoxylin and eosin stained and re-reviewed by a uropathologist to standardise and provide a contemporary histopathological assessment including cribriform and intraductal carcinoma of the prostate (IDCP). Results were reported according to the ISUP guidelines [14] and structured reporting guidelines of the Royal College of Pathologists of Australasia (RCPA). 

### 2.4. Mutation Detection

For each prostate cancer index case, 2.5 ug of gDNA underwent an 84-hereditary-cancer-gene panel screening (https://www.invitae.com/en/providers/test-catalog/test-01101 (accessed on 31 March 2021)). Variant classification was undertaken according to the five-tier ENIGMA system (http://www.enigmaconsortiumorg/ (accessed on 31 March 2021)). C1/2 variants were not reported. Relatives (male and female, cancer-affected and unaffected) of index cases in whom a C4/5 clinically notifiable, non-*BRCA* pathogenic variant was identified underwent segregation analysis to determine family-specific mutation status and identify obligate carriers. 

Prostate cancer index cases with no C4/5 variants detected and their male relatives (majority genetically unscreened) with verified prostate cancer formed the *BRCAX* control cohort (*n* = 111). Clinical characteristics of the PCOR (*n* = 39,953) and a *BRCA2* cohort (*n* = 40) [16] were obtained for comparison. PCOR is a whole-population (Australia and New Zealand) prostate cancer registry [17]. 

### 2.5. Statistical Analysis

Descriptive statistical methods defined the clinical characteristics of each gene. Prostate-cancer-specific survival for *ATM* mutation carriers and the *BRCAX* cohort were analysed using the Kaplan–Meier method and compared using log-rank (LR) test. Odds ratios (OR) were calculated using Fisher’s exact test to compare clinical characteristics of *ATM* and *HOXB13* mutations, and the *BRCAX* cohort. This analysis was not performed for *CHEK2* carriers due to low carrier numbers. Student’s *t*-test was used to evaluate differences between C3 mutation carriers and non-carriers within the *BRCAX* group. These analyses were performed using Stata 16.0.

A modified segregation analysis was used to obtain an estimate of prostate cancer risk (hazard ratio, HR) for each gene separately (*ATM*, *CHEK2*), based on genotyping of family members using MENDEL version 3.2. Estimates were adjusted for clinic-based ascertainment using the retrospective likelihood method [18]. For each gene, the cumulative risk (penetrance) to a given age was calculated as one minus the exponential of minus the cumulative incidence for carriers, which itself was the sum from age zero to the given age of the estimated HR multiplied by the non-carriers’ prostate cancer age-specific incidence [19]. Non-carrier incidences were set equal to the age-specific population incidence rates for Australia in the period 1998–2002 [20]. The HR was assumed to be constant across all ages, and all ages were truncated at 80 years. *p*-values were two-sided and based on the likelihood ratio test, and a threshold of 0.05 was used to define statistical significance.

## 3. Results

Ninety-four prostate cancer index cases were screened, yielding twenty-two (23%) C4/5 mutations across ten different genes. Fourteen of twenty-two (64%) mutations were from multi-case breast/ovarian cancer families and eight of twenty-two (36%) from multi-case prostate cancer families. Six of twenty-two variants were not clinically notifiable according to kConFab clinical notification protocol (*n* = 1 *BARD1*, *NBN*, *RECQL4*, *WRN*; *n* = 2 *PTCH1*). Seven of twenty-two variants were newly identified in *BRCA1* (*n* = 3) and *BRCA2* (*n* = 4) genes. Nine of twenty-two variants were identified in *ATM* (*n* = 4), *CHEK2* (*n* = 2) and *HOXB13G84E* (*n* = 3). gDNA segregation analysis confirmed a further six prostate-cancer-affected relatives as carriers; a final cohort for analysis contained fifteen patients (*ATM n* = 9, *CHEK2 n* = 2 and *HOXB13G84E n* = 4). Table 1 shows a summary of gene nomenclature, prostate-cancer-affected carriers and non-carriers of the family-specific mutation for each gene and recruitment stream (multi-case breast/ovarian and prostate cancer families). Variants were unique to individual families except the *HOXB13G84E* variant which was found in three families. 

### 3.1. Clinical Phenotype of Germline Mutation Carriers

#### 3.1.1. ATM Mutation Carriers

**Figure 1:**
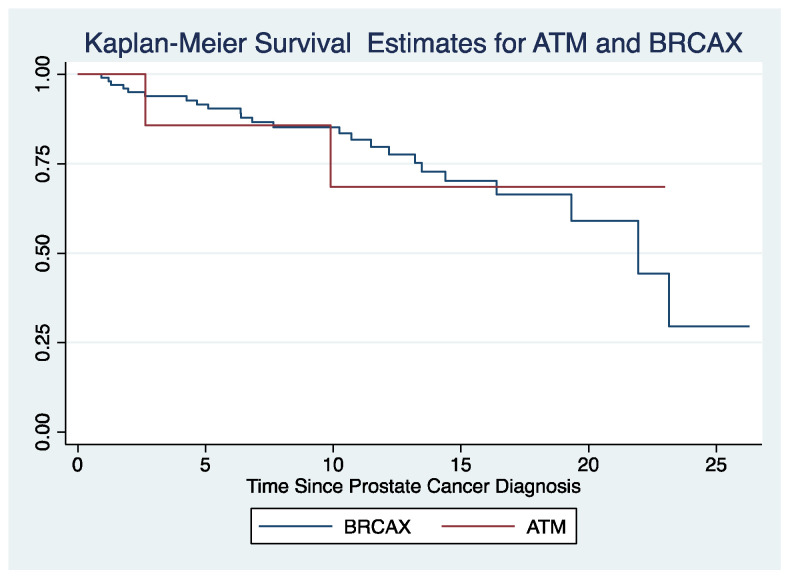
Survival time in years following prostate cancer diagnosis; red line represents survival of *ATM* mutation carriers following diagnosis, blue line represents survival of *BRCAX* cohort patients.

There were nine *ATM* mutation carriers identified with a median age at diagnosis of 65 years (mean 65, range 42–90) (Table 2). More than half (56%) of *ATM* mutation carriers presented with a PSA in the intermediate to high-risk range of >10 nm/mL. Eight *ATM* mutation carriers underwent treatment with curative intent (radiation *n* = 3, surgery *n* = 5). There were three deaths; two prostate-cancer-specific and one from myelodysplastic syndrome (MDS). The probability of prostate cancer-specific survival at five and at ten years post diagnosis was 86% (CI 33–98%) and 69% (CI 21–91%), respectively. The LR test for equality of survivor functions comparing *ATM* carriers and the *BRCAX* group was not significant *p* = 0.95 (Figure 1). Most *ATM* carriers had GG 5 disease (*n* = 3, 33.3%), advanced stage at diagnosis (*n* = 3, 33.3%) and high-grade architectural features (IDCP, cribriform structure) on pathology review (*n* = 4, 44.4%) (Table 2). One *ATM* carrier diagnosed at 42 years was stratified to the low-risk D’Amico group. The estimated HR for prostate cancer associated with *ATM* mutations was 3.5 (*p* = 0.11, 95% CI 0.69–18.2). Cumulative risks of prostate cancer approached 6% at age 50 and 20% by age 70 (Table 3). Similar to the *BRCAX* cohort, *ATM* carriers were associated with high-risk D’Amico classification and high-grade pathology (GG 4–5) with no significant statistical differences in clinical phenotype identified when comparing the two groups.

#### 3.1.2. CHEK2 Mutation Carriers

Two *CHEK2* mutation carriers were identified who were diagnosed with prostate cancer at 80 and 56 years (Table 2). The former was diagnosed with Stage T4, GG 5 prostate cancer with IDCP after a cystoprostatectomy for concurrent primary bladder cancer. His PSA fluctuated between 9–24 for five years prior; a biopsy had not been undertaken due to comorbidities and preferences with survival <1 year following surgery. The latter was diagnosed with Stage pT2c, GG 2 disease following radical prostatectomy (D’Amico high-risk) with survival > 15 years. His cancer history included primary bilateral breast cancer (Table 2). Both patients demonstrated high-grade pathology and advanced stage at diagnosis. The estimated HR for prostate cancer associated with *CHEK2* mutations was 8.6 (*p* = 0.29, 95% CI 0.40–182). The cumulative risk of prostate cancer for *CHEK2* mutation carriers approached 1.2% at age 50 and 48% by age 70 (Table 3).

#### 3.1.3. HOXB13G84E Mutation Carriers

There were four patients identified as *HOXB13G84E* mutation carriers who were identified with prostate cancer at ~66 years (mean 62, range 47–69) (Table 2). One prostate-cancer-specific death and one non-prostate-cancer-related death occurred in this group, both 25 years post diagnosis. Two carriers remain alive 13 and 12 years post diagnosis. All patients in this group underwent treatment with curative intent (surgery *n* = 3, radiation *n* = 1) (Table 2). One primary breast cancer was identified. *HOXB13G84E* carriers had GG 1–2 disease and were stratified to D’Amico low–intermediate risk, except one patient diagnosed with stage pT3a disease. PSA at diagnosis was <10 ng/mL for all participants. Clinical phenotype was not significantly different for *HOXB13* carriers compared with the *BRCAX* group when comparing low-risk D’Amico (*p* = 0.3), patients with Grade Group 1 and 2 disease (*p* = 0.3), PSA (*p* = 0.5), age at death (*p* = 0.1), age at diagnosis (*p* = 0.8) and prostate-cancer-specific death (*p* = 0.4).

### 3.2. BRCAX Cohort Analysis

Within the cohort of 111 prostate cancer patients categorised as *BRCAX* (i.e., confirmed non-mutation carriers), clinical characteristics of C3 variant carriers (*n* = 39, Supplementary Table S1) and non-carriers (*n* = 72) were not significantly different. Therefore, the *BRCAX* cohort analysis combined these sub-groups. Mean PSA at diagnosis was 13.7 ng/mL. Twenty-four prostate-cancer-specific deaths were recorded; the probability of prostate-cancer-related mortality was 21.6% (Supplementary Figure S1). Median follow-up time was 8.6 years (mean 9.5, range 1 month to 26.3 years). A total of 64.4% (*n* = 67) of patients underwent curative treatment with radical prostatectomy. Other verified primary cancers were identified in 16.7% of patients (Supplementary Table S2). The *BRCAX* group had a lower median age at diagnosis (62 vs. 68) and a higher percentage of GG 4–5 disease (30.6% vs. 25%) compared with the PCOR cohort [17]. In the *BRCAX* group, 53.2% of subjects were stratified as D’Amico high risk, compared with 25% of the PCOR cohort (Table 4, Supplementary Figure S2).

## 4. Discussion

This study demonstrates the clinical and pathological features of 15 prostate cancer patients who carry rare, pathogenic non-BRCA germline variants. Thirteen of fifteen cancers were classified as D’Amico high risk (*n* = 9, 60%) or intermediate risk (*n* = 4, 26.7%). Phenotypic differences comparing variant carriers and non-carriers in this cohort were likely diminished by the high-risk clinical features of the BRCAX group [1]. We have previously demonstrated that men with a strong family history of prostate cancer are at increased risk of developing clinically significant and aggressive disease [16]. Although germline mutations represent one mechanism through which this can occur, other mechanisms such as epigenetic gene silencing and hapolinsufficiency are also being investigated in the BRCAX cohort to explore why they demonstrate high-risk features of disease similar to the mutation-carrier groups. 

ATM and CHEK2 both appear to be less penetrant than BRCA2 genes [10,21,22]. Broader panel testing than has previously been applied to families resulted in improved identification of mutations. This is demonstrated by nine patients with clinically notifiable, pathogenic variants, seven of which were not previously detected using a breast/ovarian-cancer-gene panel when testing affected female relatives.

Germline mutations in *BRCA2*, *ATM*, *MLH1*, *PMS2*, *CHEK2* and *HOXB13* have been identified in association with prostate cancer risk [11,21,22], and carriers of germline *MSH2* and *MSH6* mutations were found to have higher prostate cancer incidence than age-matched controls (4.3% vs. 3%, respectively) [21,23]. These genes combined account for only 15% of familial prostate cancer predisposition [21,24]. Genetic testing recommendations differ between guidelines due to varying levels of evidence for rarer variants [25]. Guidelines must balance the need for cost-effective recommendations within the scope of current evidence, acknowledging that carriers of rarer variants may remain unidentified. Guidelines also currently emphasise family history of breast/ovarian cancers, rather than multi-case prostate cancer history [26]. One study found that 37% of prostate-cancer-affected pathogenic mutation carriers would not have qualified for genetic testing under the NCCN genetic/familial breast and ovarian guidelines for patients with prostate cancer, based on self-reported family histories [12]. Although some testing occurs at the discretion of practitioners, this cannot be guaranteed where funding and insurance will not cover costs [7,27]. Guidelines for broader gene panel testing based on prostate cancer family history alone are recommended [26]. 

*ATM* mutations and high-risk prostate cancer have been associated in some studies through screening of patients with advanced disease [28,29], although *BRCA2* remains the only gene in which pathogenic variants have been consistently linked to aggressive prostate cancer. In contrast to our findings, the PRACTICAL consortium investigated a pooled cohort of germline *ATM* mutation carriers, concluding that although they were associated with younger age of prostate cancer onset, variants did not conclusively predispose carriers to more aggressive prostate cancer phenotypes [10]. Conversely, a pooled analysis of loss of function (LoF) mutations, including two *ATM* mutations, concluded that even after adjusting for the inclusion of *BRCA2* mutations in the cohort, the association between poor prognosis, aggressive disease and LoF mutations in the *ATM* gene remained [30]. Testing criteria for therapeutically significant somatic and/or germline mutations such as *ATM* has been recommended following evidence that *ATM* pathogenic mutations are associated with efficacy of gene-targeted therapies and early age of prostate cancer onset [10,31]. Therefore, although small, our cohort contributes significantly to the current literature correlating *ATM* mutation carriers and phenotype. Compared with our previously published *BRCA2* cohort [16], *ATM* mutation carriers were diagnosed at a similar mean age (65 vs. 65.9) with similar PSA levels (*ATM:* 55.6% > 10 ng/mL, *BRCA2:* 35% > 10 ng/mL). Both carrier groups had a high proportion of GG 4–5 disease (*ATM* 33.3%, *BRCA2* 65.8%). Stage and PSA at diagnosis resulted in 66.7% of *ATM* carriers and 79.5% of *BRCA2* carriers being classified as D’Amico high risk. Only one *ATM* carrier was considered low risk. However, his age at diagnosis (42 years) and family history (other related carriers diagnosed at age 49 and 61) suggest a highly penetrant variant. Importantly, *ATM* mutations were detected in prostate cancer index cases from two families in which close relatives with breast/ovarian cancer had previously undergone limited *ATM* screening for the *7271* variant. In addition, two *ATM* mutations were identified in screening-naïve families with no cancer-affected female relatives. These cases support prostate-specific-gene panel testing for patients from multi-case cancer families independent of female relatives.

*CHEK2* carriers in this cohort also demonstrated high-risk features of prostate cancer. Brandao et al. recently evaluated the role of the *CHEK2* c.349A > G variant in prostate cancer development, with results supporting its candidacy as a founder mutation associated with early onset familial prostate cancer [22]. Due to its founder variant status, Brandao et al. assert that *CHEK2* may be a cost-effective addition to screening panels in select multi-case prostate cancer families [22]. *CHEK2* was also associated with aggressive prostate cancer in the pooled analysis of (LoF) mutations conducted by Leongamornlert et al., which included two carriers of *CHEK2* frameshift mutations [30]. Our cohort therefore contributes to evidence of prostate cancer predisposition for *CHEK2* carriers and a relationship with aggressive disease, although larger numbers are required to solidify this association [22].

*HOXB13G84E*-associated prostate cancer demonstrated lower-risk clinical features compared with other variants. This is in keeping with the clinical profile of *HOXB13* carriers previously described [5], although notably *HOXB13* carriers in our cohort were not associated with younger age of onset compared with non-carriers and the cohort described by Ewing et al. [32]. This is an important contribution to the prostate cancer profile of the *HOXB13G84E* variant which is significantly associated with hereditary prostate cancer development.

A limitation of our study was the assumption that if the prostate cancer index case did not carry a C4-5 mutation, then the other prostate cancer cases in that family did not either. This could have affected the clinical characteristics seen in the *BRCAX* group and would require testing of all family members to provide confirmation. C3 variants were also common (35%) in the *BRCAX* group. Their association with prostate cancer heritability and risk of aggressive disease is unknown, although Mersch et al. reported that of those with sufficient evidence to be reclassified (7.7%), 91% were reclassified as benign [33]. Despite our small carrier cohort of novel mutations, the accompanying clinical information is highly informative in elucidating the clinical phenotype associated with these pathogenic variants [34]. 

## 5. Conclusions

This study contributes to the correlation of rare genetic variants with clinically significant prostate cancer. Incorporation of gene panel testing into clinical practice will allow clinicians to identify patients with a family history of prostate cancer who are at risk of aggressive disease. Confirmation of mutation status can facilitate intensive treatment rather than active surveillance for carriers. Other benefits include early disease detection and access to novel therapies. Recruitment of additional multi-case prostate cancer families to solidify the clinical validity and utility of multi-gene panel testing for these variants in patients with familial prostate cancer is required. Through broader panel testing, changes in clinical practice and clinical outcomes are realisable for this high-risk cohort.

## Figures and Tables

**Table 1:** Gene nomenclature, affected families, carriers and non-carriers with prostate cancer (PCa) within each family and kConFab recruitment stream.

Gene	Nomenclature	Affected Families (n)	Carriers with PCa (n)	Non-Carriers with PCa (n)	Recruitment Stream
*ATM*	c.6115G > A; p.Glu2039Lys	1	2	0	Breast/ovarian
	c.8266A > T; p.Lys2756Ter	1	2	0	Breast/ovarian
	c.8395_8404del	1	2	1	Prostate
	c.3712_3716del; p.Leu1238Lysfs 6	1	3	0	Prostate
*CHEK2*	c.349A > G; p.Arg117Gly	1	1	0	Breast/ovarian
	c.499G > A; p.Gly167Arg	1	1	0	Breast/ovarian
*HOXB13*	G84E	3	4	0	Breast/ovarian
*PTCH1*	c.3850C > T; p.Gln1284	1	1	0	Breast/ovarian
	c.290dup; p.Asn97Lysfs 43	1	1	0	Breast/ovarian
*BARD1*	c.1921C > T; p.Arg641	1	2	0	Breast/ovarian
*NBN*	c.217A>T; p.Lys73	1	1	0	Breast/ovarian
*RECQL4*	c.2269C > T; p.Gln757	1	1	1	Breast/ovarian
*WRN*	c.3030_3033del; p.Thr1011Argfs 11	1	1		Prostate
*BRCA1*	c.2612insT; p.PHe872Valsfs 31	1	2	0	Breast/ovarian
	c.2864C > A; p.Ser955	1	0	0	Prostate
	c.547+1G > T (Splice donor)	1	2	0	Prostate
*BRCA2*	c.67+1G > T; (Splice donor)	1	1	0	Breast/ovarian
	c.7266T > A; p.Cys2422	1	2	0	Prostate
	c.5909C > A; p.Ser1970	1	1	0	Prostate
	c.8756G > A; p.Gly2919Asp	1	1	0	Prostate

**Table 2:** Clinical characteristics for *ATM*, *CHEK2*, and *HOXB13* germline mutations.

*ATM* Mutation Carriers									
Age at Diagnosis	PSA ^1^	Grade Group	Stage	Architecture ^2^	D’Amico	Primary Treatment	Age at Death	Cause of Death	Age at Diagnosis of Other Primary Malignancy
78	13.6	-	-	-	Int	Radiation	87	PCa	-
90	210	5	T1c	No	High	Non-curative	93	PCa	-
57	160	-	-	-	High	Radiation	75	MDS ^3^	70, MDS
65	20	5	T1c	Cribriform	High	Radiation	-	-	66, Renal
75	5	3	T3aN1	Cribriform	High	Prostatectomy	-	-	
68	12	5	T3b	IDCP	High	Prostatectomy	-	-	-
49	2.6	3	T2a	IDCP, Cribriform	Int	Prostatectomy	-	-	-
42	6.9	1	-		Low	Prostatectomy	-	-	-
61	12	2	T2c	-	High	Prostatectomy	-	-	83, Lung, Melanoma
***CHEK2* Mutation Carriers**									
80	-	5	T4	-	High	Prostatectomy	81	Cardiac	78, Bladder, Colon
56	8.8	2	T2c	-	High	Prostatectomy	-	-	47, Breast
***HOXB13G84E* Mutation Carriers**									
68	6.7	1	T1c		Low	Radiation	92	-	80, Breast
69	5.3	2	T2N0		Int	Prostatectomy	-	-	-
47	3.8	2	T3a		High	Prostatectomy	-	-	-
64	-	2	T2a		Int	-	89	PCa	-

^1^ PSA immediately prior to undergoing diagnostic biopsy; ^2^ High-risk pathology features identified within prostatic adenocarcinoma; Abbreviations: Myelodysplastic syndrome (MDS), D’Amico Intermediate-risk (Int), intraductal carcinoma of the prostate (IDCP).

**Table 3:** Cumulative Risk of Prostate Cancer Diagnosis for *ATM* and *CHEK2* mutation carriers.

Percentage Cumulative Risk (95% CI) of Prostate Cancer Diagnosis		
Age (Years)	*ATM* Mutation Carriers	*CHEK2* Mutation Carriers
30	0 (0–0)	0 (0–0.1)
40	0 (0–0)	0 (0–0.4)
50	0.5 (0.1–2.5)	1.2 (0.1–22.4)
60	5.7 (1.1–25.9)	13.2 (0.7–95)
70	20.6 (4.4–69.4)	42.8 (2.6–100)
80	40.9 (9.7–93.3)	72.1 (5.8–100)

**Table 4:** Clinical features of the *BRCAX* cohort, PCOR cohort and *BRCA2* cohort.

	*BRCAX* (*n* = 111)	PCOR1 (*n* = 39,953)	*BRCA2* (*n* = 40) ^1^
Age at Diagnosis	62.7 (mean) 62 (median) 42–84 (range) 9.7 (SE)	68 (median)	65.9 (mean) 64.95 (median)
PSA pre-diagnosis, *n* (%)			
<4 ng/mL	17 (15.3%)	955 (13%)	1 (2.5%)
4–10 ng/mL	43 (38.7%)	4111 (56%)	10 (25%)
10+ ng/mL	25 (22.5%)	2225 (31%)	14 (35%)
Unknown	26 (23.4%)	1067 (14%)	11 (27.5%)
ISUP Grade Group at Diagnosis, *n* (%)			
1	19 (17.1%)	2115 (26%)	2 (5.3%)
2	32 (28.8%)	2606 (32%)	11 (28.9%)
3	23 (20.7%)	11,380 (7%)	
4 & 5	34 (30.6%)	2002 (25%)	25 (65.8%)
Unknown	3 (2.7%)	255 (4%)	-
D’Amico Risk Group at Diagnosis			
Low	21 (18.9%)	1492 (20%)	3 (7.7%)
Intermediate	31 (27.9%)	3454 (46%)	5 (12.8%)
High or very high	59 (53.2%)	1832 (25%)	31 (79.5%)
Survival Status			
Deceased *n* (%)	30 (27%)	-	23 (57.5)
PCa-specific death (*n*)	24 (21.6%)	-	21 (52.5%)
Mean, median years of follow up from diagnosis (range)	9.5, 8.6 (0.2–26.3)	–	4.6 years (high risk)9 years (int. risk)
Mean, median duration to death (years)	9.9, 8.8 (0.9–26.3)	–	4.5 (mean)
PCa-specific survival estimate	91% 5-yr (84–96)	95% 5-yr (2012–2016) ^2^	33% 15-yr (int. risk)
PCa-specific survival estimate	85% 10-yr (76–91)	91.3% 10-yr (2011–2015) ^3^	0% 15-yr (high risk)

^1^*BRCA2* cohort previously described in Bolton et al. *BJU Int.* **2015**, *116*, 207–212 [17]. ^2^ https://www.canceraustralia.gov.au/cancer-types/prostate-cancer/statistics (accessed on 10 September 2021); ^3^ https://ncci.canceraustralia.gov.au/outcomes/relative-survival-rate/10-year-relative-survival (accessed on 10 September 2021).

## Data Availability

The dataset can be made available upon request.

## Supplementary Materials

The following supporting information can be downloaded at: https://www.mdpi.com/article/10.3390/cancers14153623/s1, Figure S1: Survival time in years for BRCAX cohort patients, following a diagnosis of prostate cancer; Figure S2: Kaplan Meier curve showing prostate cancer-specific survival of patients from the PCOR cohort following a diagnosis of prostate cancer; Table S1: C3 variant nomenclature identified in the *BRCAX* cohort; Table S2: Non-Prostate Primary Cancers verified among the *BRCAX* cohort.
