# Supplementary material for: Novel Germline Mutations in a Cohort of Men with Familial Prostate Cancer
Source: Cancers (Basel). 2022 Jul 26;14(15):3623. doi: 10.3390/cancers14153623 (PMC9332384; doi:10.3390/cancers14153623)
Supplement: Supplementary file 1 [file cancers-14-03623-s001.zip › cancers-1805198-supplementary.pdf]

Supplementary Table S1: C3 variant nomenclature identified in the *BRCAX* cohort

Gene	Variant Nomenclature
ALK	c.2488A>T; (p.Met830Leu)
	c.2489T>C ; (p.Met830Thr)[8]
APC	c.4963A>G; (p.Thr1655Ala)[5]
	c.5282A>C; (p.Asn1761Thr)[5]
	c.5939A>G ; (p.Asn1980Ser)[NBN]
ATM	c.7399G>A ; (p.Val2467Ile)
	c.5042T>C ; (p.Ile1681Thr)
	c.5190A>G ; (Silent)
AXIN2	c.2405+4A>G ; (Intronic)
	c.544A>G, p.Met182Val
BAP1	c.1810G>A ; (p.Val604Met)
BLM	c.968A>G; (p.Lys323Arg)[6]
BMPR1A	c.751G>T ; (p.Gly251Cys)
BRCA1	c.-19-235G>A; IVS1-235A>G[2]
BRIP1	c.2563C>T ; (p.Arg855Cys)
CDH1	c.1435G>A ; (p.Asp479Asn)
	c.2635G>A; p.Gly879Ser exon 16
CDKN1B	c.407A>G; (p.Asp136Gly)[1]
CHEK2	c.1175C>T; (p.Ala392Val)
DICER1	c.1561A>G ; (p.Ile521Val)
DIS3L2	c.1337G>T; p.Arg446Met
EGFR	c.3629C>T; (p.Ala1210Val)
FH	c.379G>T; p.Val127Leu[12]
MSH6	c.2641delinsAAAA (p.Gly881delinsLysSer)
	c.1538T>C ; (p.Ile513Thr)[BRCA2]
MSH2	c.1313C>G; p.Thr438Ser
MUTYH	c.719C>T ; (p.Ala240Val)[10]
	c.349-4A>T ; (Intronic)
PALB2	c.2674G>A; (p.Glu892Lys)[4]
	c.2417C>T; (p.Pro806Leu)
	c.3494C>G; (p.Ser1165Trp)
	c.2816T>G p.Leu939Trp
PDGFRA	c.2711C>T ; (p.Thr904Ile)
POLD1	c.245C>T; (p.Pro82Leu)
	c.3221G>A; (p.Arg1074Gln)
	c.3136G>T; (p.Ala1046Ser)[9]
POLE	c.1999A>C; (p.Met667Leu)[4]
	C3/c.1357C>G ; (p.Gln453Glu)[6]
	c.1A>T; (p.Met1?)[8]

	c.4736G>A (p.Arg1579His)[11]
POT1	c.670G>A; (p.Asp224Asn)[2]
RAD51C	c.904+34T>C; (rs28363318)[1, 3]
	c.536A>G; (p.His179Arg)
RB1	c.48C>A ; (Silent)
RET	c.1878G>A; (Silent)[10]
RUNX1	c.613+2dup ; (Splice site)[7]
SMARCA4	c.1497G>T; (p.Lys499Asn)[7]
	c.4981G>A ; (p.Asp1661Asn)[9]
TSC2	c.4393G>A (p.Asp1465Asn)[11]
WRN	c.334T>C; (p.Phe112Leu)
	c.229G>C; (p.Asp77His)[3]
	c.4165A>G; p.Lys1389Glu[12]

**some men carried more than one variant; different variants carried by the same patient are denoted with matching superscript eg. a single patient carried both variants denoted by [1], another individual carried both variants denoted by [2].*

Supplementary Table S2: Non-Prostate Primary Cancers verified among the *BRCAX* cohort

***BRCAX* Group Other Verified Primary Cancers n, (%)**	
Breast	1 (0.93%)
Colon/colorectal	4 (3.7%)
Melanoma	8 (6.5%)
Blood (MDS/lymphoma)	3 (2.8%)
Bladder	2 (0.93%)
Lung	2 (1.85%)

Supplementary Figure S1. Survival time in years for *BRCAX* cohort patients, following a diagnosis of prostate cancer. Censorship from the study is indicated with vertical lines.

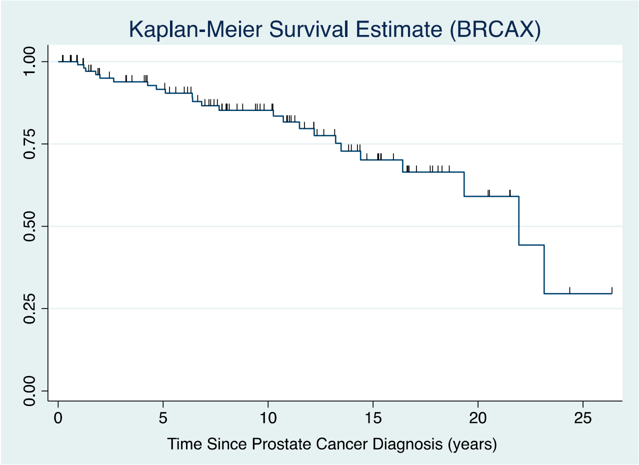

Supplementary Figure S2. Kaplan Meier curve showing prostate cancer-specific survival of patients from the PCOR cohort following a diagnosis of prostate cancer. The cohort was subdivided by ISUP Grade with worse survival outcomes evident for patients with ISUP 5 disease (bottom line) and ISUP 4 disease (second from bottom) compared to patients with lower grade disease[17]

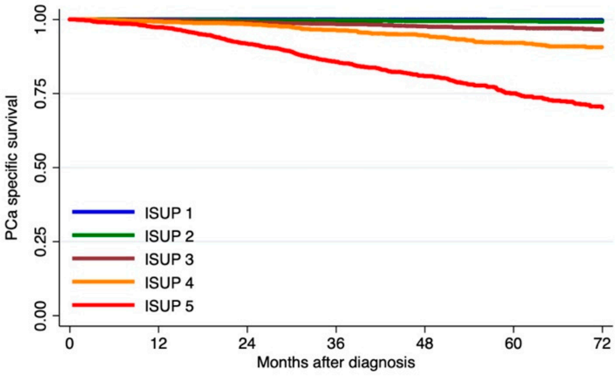

ISUP 1	5711	(0)	5084	(0)	4255	(1)	3474	(1)	2740	(2)	2008	(1)	1377
ISUP 2	6653	(7)	5673	(7)	4561	(4)	3501	(4)	2652	(2)	1949	(2)	1237
ISUP 3	3312	(10)	2826	(13)	2287	(14)	1709	(12)	1260	(4)	872	(5)	518
ISUP 4	2019	(13)	1753	(14)	1437	(26)	1091	(19)	811	(18)	574	(8)	360
ISUP 5	2664	(63)	2155	(109)	1599	(93)	1140	(56)	833	(49)	495	(26)	285
